# Polyphosphate kinase deletion increases laboratory productivity in cyanobacteria

**DOI:** 10.3389/fpls.2024.1342496

**Published:** 2024-02-07

**Authors:** Jacob Sebesta, Michael Cantrell, Eric Schaedig, Harvey J. M. Hou, Colleen Pastore, Katherine J. Chou, Wei Xiong, Michael T. Guarnieri, Jianping Yu

**Affiliations:** ^1^ Biosciences Center, National Renewable Energy Laboratory, Golden, CO, United States; ^2^ Laboratory of Forensic Analysis and Photosynthesis, Department of Physical Sciences, Alabama State University, Montgomery, AL, United States

**Keywords:** cyanobacteria, energy regulation, polyphosphate, biocontainment, ethylene

## Abstract

Identification and manipulation of cellular energy regulation mechanisms may be a strategy to increase productivity in photosynthetic organisms. This work tests the hypothesis that polyphosphate synthesis and degradation play a role in energy management by storing or dissipating energy in the form of ATP. A polyphosphate kinase (*ppk*) knock-out strain unable to synthesize polyphosphate was generated in the cyanobacterium *Synechocystis* sp. PCC 6803. This mutant strain demonstrated higher ATP levels and faster growth than the wildtype strain in high-carbon conditions and had a growth defect under multiple stress conditions. In a strain that combined *ppk* deletion with heterologous expression of ethylene-forming enzyme, higher ethylene productivity was observed than in the wildtype background. These results support the role of polyphosphate synthesis and degradation as an energy regulation mechanism and suggest that such mechanisms may be effective targets in biocontainment design.

## 1 Introduction

All organisms experience fluctuations in environmental conditions which impact their metabolism. Cells have adapted various mechanisms for maintaining growth despite fluctuations in nutrient concentrations, temperature, environmental stresses, and biological stresses. Microalgae use several energy management mechanisms for tolerating variations in light intensity and nutrient availability (Van Thor et al., 1998; Bailey and Grossman, 2008; Berera et al., 2009; Demmig-Adams et al., 2014; Montgomery, 2014). Cells must store energy for dark periods and dissipate excess energy when the light intensity exceeds the amount that can be utilized. For example, the flavodiiron proteins, Flv1 and Flv3, are needed to protect photosystems and sustain growth in fluctuating light intensities (Allahverdiyeva et al., 2013). Deletion of certain genes responsible for energy management may result in reduced fitness in natural environmental conditions.

Large-scale cultivation of genetically modified microalgae carries risks associated with the possibility of unintentional release of the organism into the environment. Escape of engineered cells into the environment may have negative impacts ranging from horizontal gene transfer to perturbation of ecosystems resulting from proliferation or persistence of those cells. Cultivated microalgae including cyanobacteria may easily access the natural environment because they are likely to be grown outdoors to utilize sunlight. To prevent environmental impacts arising due to escape, a variety of biocontainment strategies have been developed recently (Gressel et al., 2013; Gressel et al., 2014; Arnolds et al., 2021; Sebesta et al., 2022). Typically, these strategies rely on the conditional expression of a toxic protein which remains repressed in normal cultivation but is expressed upon escape. Many such strategies have been demonstrated to effectively kill cells, yet these systems frequently have reduced productivity in their host organisms either due to leaky expression of the toxin, or due to the metabolic burden of expressing multiple regulatory components (RNA and proteins) to carefully control expression of the toxin (Liu and Curtiss, 2009; Zhou et al., 2019). Genetic constructs encoding toxins are also susceptible to inactivation by mutations (Knudsen and Karlström, 1991; Molin et al., 1993). Deletion of genes that are essential in common environmental conditions but not needed in controlled lab conditions is a promising alternative that has been shown to be effective. For example, deletion of carboxysome genes from *Synechococcus* sp. PCC 7002 created a high carbon-dependent strain (Clark et al., 2018). Metabolic engineering entails the creation of specialist strains of microbes capable of producing high quantities of valuable chemicals in a defined cultivation system. Such strains should also be designed to have reduced fitness in natural environments.

**Figure 1:**
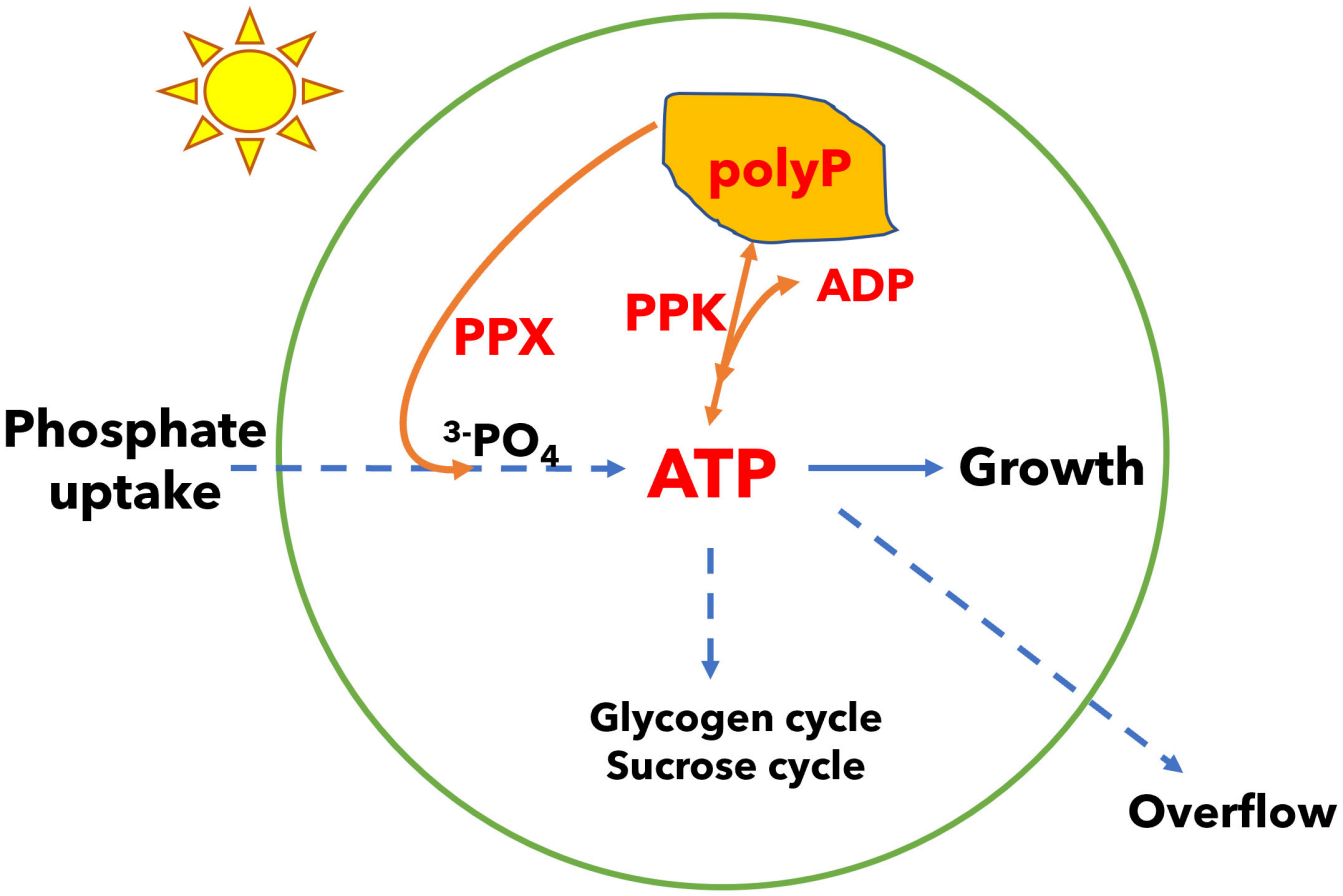
A model of energy regulation in *Synechocystis* by polyphosphate (polyP) synthesis and degradation. Phosphate is incorporated in adenosine triphosphate (ATP). Polyphosphate kinase (PPK) can reversibly transfer phosphate from ATP to polyphosphate and may regulate the ATP/ADP ratio. Polyphosphate can also be degraded by exopolyphosphatase (PPX) in a reaction that does not recover ATP. Growth requires management of the ATP/ADP ratio (or energy charge). Cells may manage this ratio by polyphosphate synthesis and degradation, glycogen and sucrose synthesis and degradation cycles, and overflow metabolism which may all be used to dissipate energy. Organisms that capture light energy may depend on multiple energy dissipation mechanisms because light energy can fluctuate independent of the concentrations of nutrients needed for growth.

In addition to energy dissipation mechanisms in the light reactions of photosynthesis, metabolism plays a role in energy management. For example, energy and carbon may be stored in the form of glycogen and recovered to sustain metabolism in the dark and to restart photosynthesis during dark to light transitions (Holland et al., 2016; Makowka et al., 2020; Shinde et al., 2020; Tanaka et al., 2023). We have shown that cyanobacteria utilize several mechanisms in carbon metabolism including the glycogen synthesis and degradation cycle, the sucrose synthesis and degradation cycle, and overflow metabolism (the accumulation of organic acids in the medium) to dissipate excess energy in ATP and generate ADP (Cano et al., 2018; Cantrell et al., 2023). While ATP provides energy in many biosynthetic reactions, ADP is also needed for metabolism, thus ATP energy dissipation mechanisms are integral to cellular energy management (Sanz-Luque et al., 2020a). In the glycogen synthesis and degradation cycle, glucose phosphate is activated with energy input from ATP so a glucose unit can be added to glycogen polymer; when a glucose unit is released from the polymer to glucose phosphate, there is no ATP generation, so this futile cycle leads to loss of ATP (while generating ADP). Similarly, the sucrose cycle dissipates ATP energy. Loss of the glycogen cycle leads to increased energy charge (the ratio of ATP in the pool of ATP plus ADP) and triggers overflow metabolism and the accumulation of organic acids in the growth medium. In this work, we examined another possible metabolic modulator of cellular energy which consists of a polyphosphate synthesis and degradation cycle. ATP levels in the cell may be decreased (and ADP increased) by synthesis of polyphosphate via polyphosphate kinase (PPK), and ATP may be recovered by the reverse reaction. Alternatively, polyphosphate may be degraded by exopolyphosphatase (PPX) without recovery of ATP (**Figure 1**).

To investigate a possible role of polyphosphate in energy management and as a potential target for biocontainment, we have generated a knockout mutant of the *ppk* gene in the model cyanobacterium *Synechocystis* sp. PCC 6803 (referred to hereafter as *Synechocystis*). In other cyanobacteria and eukaryotic algae, deletion of the gene responsible for polyphosphate synthesis has resulted in higher ATP levels and reduced fitness when challenged with several different stresses (Gomez-Garcia et al., 2013; Aksoy et al., 2014; Sanz-Luque et al., 2020a). As a phosphorus storage molecule, polyphosphate may also be important for tolerance to fluctuating phosphorus concentrations, although several other roles for polyphosphates have also been proposed including involvement in stress response, divalent metal sequestration, oxidative stress protection, and protein localization (Kornberg et al., 1999; Kulaev and Kulakovskaya, 2000; Kuroda et al., 2001; Thomas and O’Shea, 2005; Zhao et al., 2008; Gross and Konieczny, 2020). In *E. coli,* polyphosphate has been shown to have a role in chelating iron providing a storage reservoir while preventing reactive oxygen species formation via the Fenton reaction (Beaufay et al., 2020). Modeling suggests that polyphosphate synthesis may also facilitate phosphate uptake by reducing the intracellular phosphate concentration and thus increasing the concentration gradient across the cell membrane (John and Flynn, 2000). We hypothesize that the Δ*ppk* mutant may show improved productivity by limiting energy loss through a possible futile cycle through PPK and PPX. In addition, we expect this strain to have reduced tolerance to environmental variations.

## 2 Methods

### 2.1 Strain generation

The *ppk* knockout plasmid (**Supplementary Figure S1**) was assembled using Gibson assembly (New England Biolabs Gibson Assembly Master Mix). The *mazF* plasmid (**Supplementary Figure S8**) was synthesized by Twist Bioscience. Ethylene forming enzyme plasmids were previously constructed (Wang et al., 2018). Transformation of *Synechocystis* was achieved by natural uptake following the protocol in Sebesta, Zimont, and Peebles (Sebesta et al., 2019). Strains were confirmed by colony PCR (**Supplementary Figures S2**, **S9**) using Taq or Phusion DNA polymerase (New England Biolabs) and PCR amplicons were sequenced by Genewiz/Azenta or plasmidsaurus.

### 2.2 Cultivation

The typical growth condition used relied on BG11 medium (Stanier et al., 1979) supplemented with 20 mM TES-NaOH, pH 7.4, and 100 mM sodium bicarbonate. Cultures were grown in shake flasks at ~40 µmol photons m^-2^ s^-1^ white light at 30°C, and 5% carbon dioxide atmosphere and shaking at 160 RPM. The moderate light condition used was the same except the light intensity was increased to approximately 200 µmol photons m^-2^ s^-1^. Low light, mixotrophic condition (LL, Mix.) used the low light intensity and BG11 medium supplemented with 5 mM glucose. Low inorganic carbon conditions (low Ci) used BG11 that omitted both sodium carbonate and sodium bicarbonate. In experiments examining the shift from high carbon to low carbon, cells were centrifuged at 6,000 x g for 6 minutes, resuspended in fresh media of the type used in the next stage of cultivation, centrifuged once more, and again resuspended in fresh media before inoculation. Optical density at 730 nm was measured in 1 mL cuvettes using a Biochrom WPA Biowave II spectrophotometer.

### 2.3 Polyphosphate measurement

Polyphosphate was quantified using DAPI fluorescent staining (Schaedig et al., 2023). 1 mL samples of cultures were collected and centrifuged to collect. Cell pellets were frozen at -80°C before processing. Pellets were resuspended in 10 mM HEPES, pH 7.4 with 0.05% (v/v) Nonidet NP-40 detergent. Cells were lysed by boiling for 10 minutes at 100°C followed by sonication on ice (Qsonica Q500 sonicator with 1.6 mm probe, 30% power, 3 s on/3 s off for 150 s). The insoluble fraction of lysate was removed by centrifugation. 200 µL of the lysed cell supernatant was transferred to a new 1.5 mL tube for removal of possibly interfering macromolecules. RNA was degraded by incubating for 10 minutes at 37°C with 100 units RNase (Ambion RNase Cocktail). DNA degradation followed with addition of 10 units DNase (Invitrogen TURBO Dnase) and incubation for 10 minutes at 37°C. Proteins were degraded by incubation at 37°C for 30 minutes following addition of Proteinase K (Roche Proteinase K). 30 µL of the digested sample was transferred to a clear bottom, black-sided 96-well plate. To each well, 30 µL of 10,000x diluted DAPI (Sigma) was added. Fluorescence was measured (excitation: 415 nm, emission: 550 nm) after 5 minutes incubation at room temperature. A standard curve was determined using polyphosphate with an average chain length of 100 phosphate units (Kerafast). Dry weight was determined by collecting 10 mL of culture on a pre-weighed glass fiber filter, drying in an oven at 60°C for more than two days, and weighing on analytical balance.

### 2.4 Energy charge measurement

Energy charge measurements utilized a Sigma-Aldrich ADP/ATP ratio assay kit (Cat. No. MAK135). 10 µL samples were transferred from shake flasks to a 96-well plate (clear bottom, white sided) placed on top of an LED light panel set to match the light intensity of the cultivation condition. The kit instructions were followed and luminescence was measured on a Tecan Infinite M200 Pro plate reader. Reported energy charge values were calculated by dividing the ATP measurement by the sum of the ATP and ADP measurements.

### 2.5 Ethylene measurement

Ethylene measurement followed the method presented by Wang et al. (2018). Cultures were grown in shake flasks with an initial OD of 0.1. When cultures reached an OD of approximately 0.5, 4 mL was transferred to 25 mL Hungate tubes and sealed. After 16 hours, 500 µL of headspace gas was injected into an Agilent 7890A gas chromatograph using TCD and a Porapak N column.

## 3 Results

### 3.1 Δ*ppk* has a higher energy charge than wildtype after re-addition of phosphate following P-starvation

**Figure 2:**
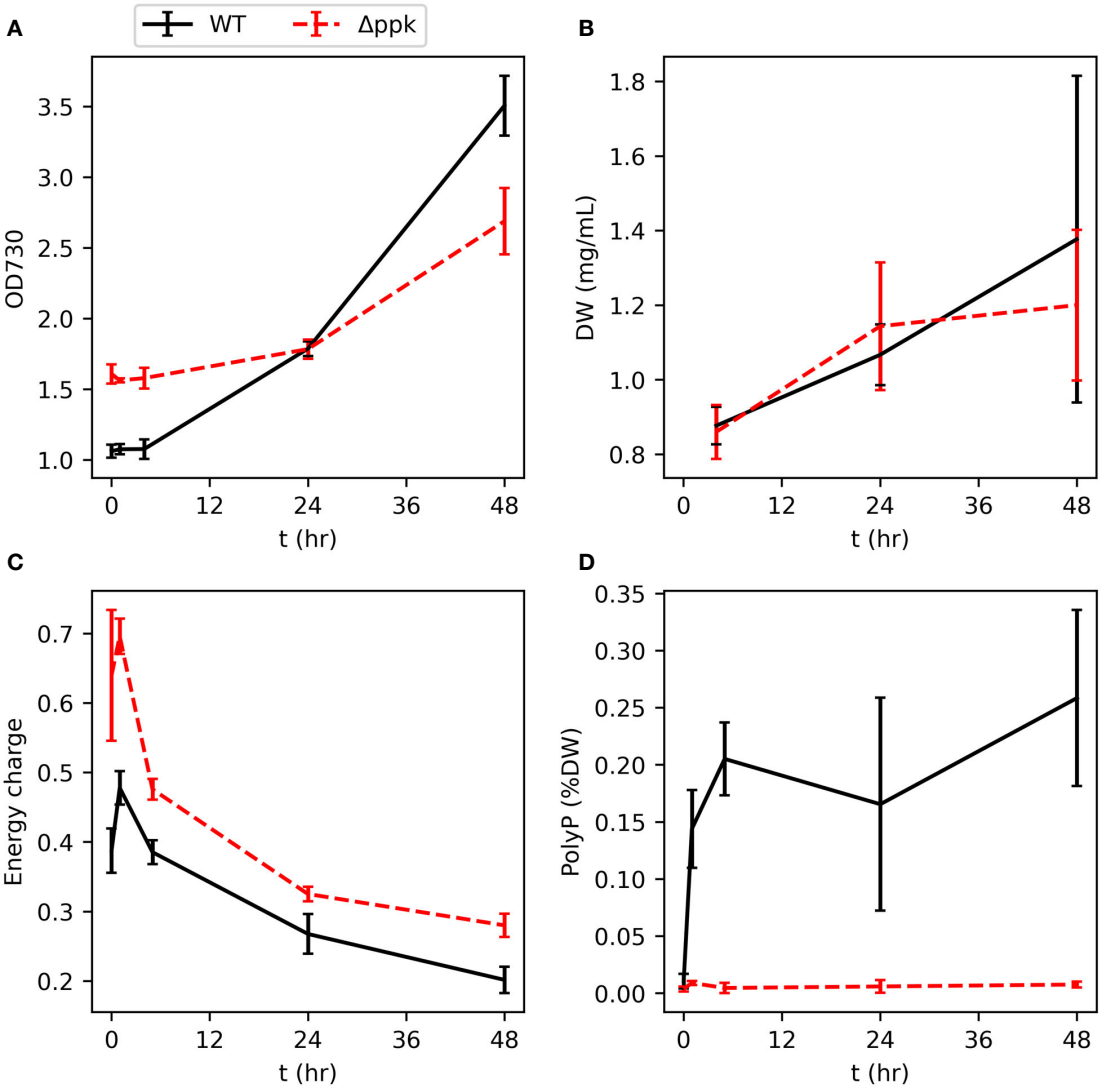
Resumption of growth of WT and Δ*ppk* when phosphate is added to phosphorus-starved cultures. **(A, B)** Dry weight (DW) and OD increases similar to WT upon re-addition of phosphate at t = 0. **(C)** The energy charge (EC) of Δ*ppk* is elevated compared with WT following phosphate re-addition. **(D)** WT rapidly accumulates polyphosphate after addition of phosphate to P-starved cultures and Δ*ppk* is incapable of polyphosphate synthesis. All error bars represent the standard deviation of three biological replicates.

We generated a *ppk* knockout strain by natural uptake of plasmid DNA which, *via* homologous recombination, replaced *ppk* with a selection marker (**Supplementary Figures S1**, **S2**). To verify the mutant’s inability to accumulate polyphosphate and examine the ability of the mutant to resume growth after phosphorus starvation, phosphate was spiked into the flasks to 175 µM (the typical concentration in BG11) following five days of cultivation in BG11 media without phosphorus source. The cultures were inoculated in the phosphorus starvation medium at the same OD. A sudden increase in phosphate concentration following phosphorus starvation typically results in “luxury uptake,” or a rapid and unusually high accumulation polyphosphate (Voronkov and Sinetova, 2019; Solovchenko et al., 2020). In green algae, this phenomenon has been characterized as resulting from the delayed transition from high affinity Pi-uptake during scarcity to the low affinity Pi-uptake typically utilized during abundance with the excess Pi stored as polyphosphate (Solovchenko et al., 2019). The wildtype (WT) and Δ*ppk* strains showed similar increases in dry weight over the following two days (**Figures 2A, B**). Different initial optical densities shown in **Figure 2A** reflect different growth during the phosphorus starvation period. Polyphosphate rapidly increased in the WT from a very low level within 1 hour (**Figure 2C**). As a percentage of cell dry weight, polyphosphate peaked after 5 hours and subsequently reduced slightly. As expected for the mutant, polyphosphate did not increase after phosphate re-addition. Furthermore, the energy charge (EC, calculated by ATP/(ATP+ADP)) was higher in the mutant than in the WT during the 48 hours following phosphate re-addition (**Figure 2D**). In both strains, EC peaked 1 hour after phosphate re-addition and then gradually decreased.

In separate experiments, we found that in our typical growth conditions (~40 µmol photons m^-2^ s^-1^, 30°C and 5% CO_2_) WT depleted phosphate more quickly from the medium than Δ*ppk* (**Supplementary Figure S3**) despite having similar growth (**Supplementary Figure S4**). Following five days of phosphorus starvation, WT had faster growth than Δ*ppk* when phosphate was added to 20 µM (~10% normal BG11 recipe) (**Supplementary Figure S5**). These data are consistent with polyphosphate having a role in phosphate uptake.

### 3.2 Δ*ppk* grows faster in high carbon conditions

**Figure 3:**
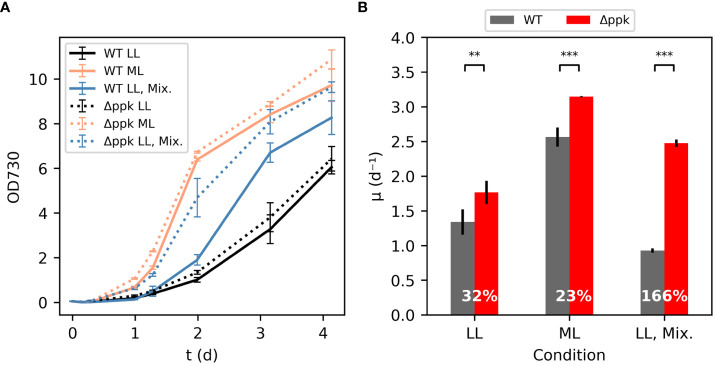
Δ*ppk* grew faster than WT in high carbon conditions. **(A)** Growth curves for WT and Δ*ppk* in low light (LL:30 µmol photons m-2 s-1, 30°C, 5% CO2 atmosphere) condition, moderate light (ML: 200 µmol photons m^-2^ s^-1^), and mixotrophic (LL-Mix.: 30 µmol photons m^-2^ s^-1^, 5 mM glucose added) conditions. **(B)** The Δ*ppk* mutant had a higher initial specific growth rate than WT (Tukey HSD test - ***p<0.001, **p<0.01). All error bars represent the standard deviation of three biological replicates.

In some conditions, the Δ*ppk* mutant grew faster than wildtype (WT) (**Figure 3**). In low light (LL) conditions, 40 µmol photons m^-2^ s^-1^ is supplied to shake flasks in a growth chamber maintained at 30°C and 5% CO_2_. In addition, the media is highly alkaline with 100 mM sodium bicarbonate and 20 mM TES-NaOH, pH 7.4 supplemented to the BG11 media. The initial growth rate of the Δ*ppk* strain was ~32% higher than the WT under the low light condition. At moderate light intensity (ML: ~200 µmol photons m^-2^ s^-1^), both strains grew much faster, and the mutant maintained some advantage over the WT in initial growth rate. In a separate experiment using ~500 µmol photons m^-2^ s^-1^, the mutant had a 26% higher initial growth rate than the WT (5.26 d^-1^ for Δ*ppk* versus 4.18 d^-1^ for WT, **Supplementary Figure S6**). In low light and mixotrophic conditions with glucose supplemented (LL, Mix.), the WT showed similar growth to the LL, autotrophic condition through the first day before the growth rate increased. In contrast, the mutant did not show such a delay in faster growth. In this condition, the mutant had an initial growth rate 166% higher than the WT.

### 3.3 Faster growth of the mutant is correlated with a higher initial energy charge

**Figure 4:**
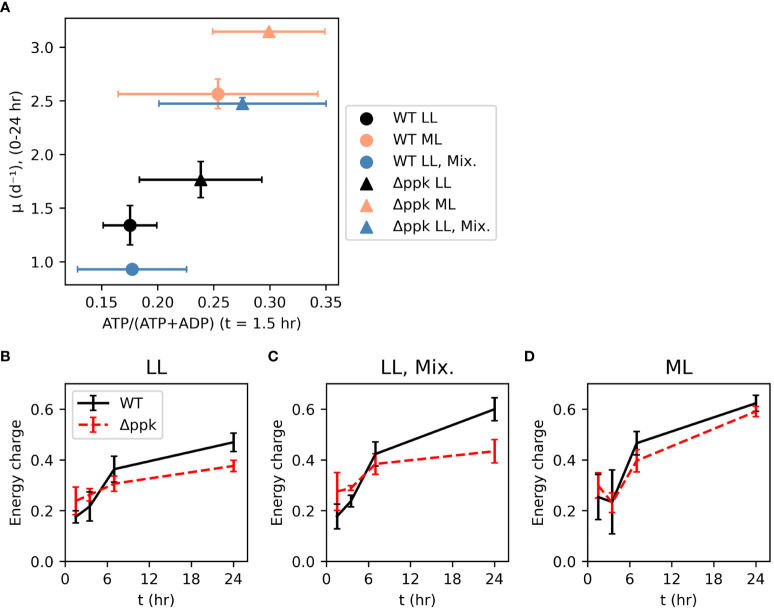
Faster growth correlates with energy charge early in growth curve and mutation of Δ*ppk* increased both energy charge and initial growth rate **(A)**. However, higher growth rates of the mutant do not depend on higher energy charge later in the growth curve [**(B)** Low light (LL: ~30 µmol photons m^-2^ s^-1^], **(C)** Mixotrophic (5 mM glucose, ~30 µmol photons m^-2^ s^-1^), **(D)** moderate light (ML: ~200 µmol photons m^-2^ s^-1^). All error bars represent the standard deviation of three biological replicates.

The energy charge (EC) was measured 1.5 hours after inoculation (**Figure 4A**). Although these measurements were noisy, a correlation was observed between this initial energy charge and the initial growth rate (between 0 and 24 hours after inoculation) across growth conditions and strains. Within each growth condition, Δ*ppk* had higher initial EC and initial growth rate, with the most dramatic increase in both parameters observed in the mixotrophic condition. The energy charge was also measured at 3.5, 7, and 24 hours after inoculation (**Figures 4B–D**). Generally, EC started around 0.2-0.3 at 1.5 hours and increased over time with a rapid increase in the first 7 hours, and a slower increase subsequently. In lower light intensity (autotrophic or mixotrophic), the mutant had slightly higher EC at 1.5 and 3.5 hours, then lower EC than WT. Mixotrophic and 200 µmol photons m^-2^ s^-1^ cultures generally had higher EC at 24 hours compared with the autotrophic 30 µmol photons m^-2^ s^-1^ cultures. Although the mutant had a higher growth rate at 24 hours than WT, at this time the EC was lower than that of WT suggesting that elevated EC may not be needed to sustain faster growth.

### 3.4 Faster growth of the Δ*ppk* mutant enables higher ethylene productivity

**Figure 5:**
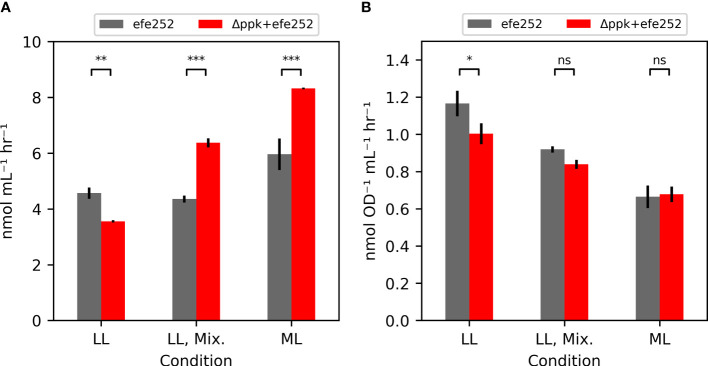
Ethylene productivity is shown in **(A)**, and OD-specific ethylene productivity in **(B)**. Ethylene production rates measured from the efe252 construct either in the WT background (WT) or combined with the *ppk* knockout. Each strain was tested under the low light (LL: 30 µmol photons m^-2^ s^-1^), mixotrophic (LL, Mix.: 30 µmol photons m^-2^ s^-1^, 5 mM glucose), and in moderate light (ML: 200 µmol photons m^-2^ s^-1^). All error bars represent the standard deviation of three biological replicates (Tukey HSD test - ***p<0.001, **p<0.01, *p<0.05, ns: not significant p>0.05).

Additional strains containing the ethylene-forming enzyme from *Pseudomonas syringae* (Wang et al., 2018), including *efe* alone (*efe*252), and *efe* in a Δ*ppk* background (Δ*ppk*+*efe*252) were generated to test the impact of biocontainment strategies on productivity (**Figure 5A**). Under low light (~30 µmol photons m^-2^ s^-1^), Δ*ppk* reduced ethylene productivity. In mixotrophic and moderate light conditions where Δ*ppk* mutant grew faster than WT, the Δ*ppk*+*efe*252 strain had higher ethylene productivity (46% and 40% higher) than *efe*252. The increase productivity may be attributable to faster growth because the OD-specific productivity in these conditions were similar (**Figure 5B**).

### 3.5 The Δ*ppk* mutant has reduced fitness in low pH and low-carbon conditions

**Figure 6:**
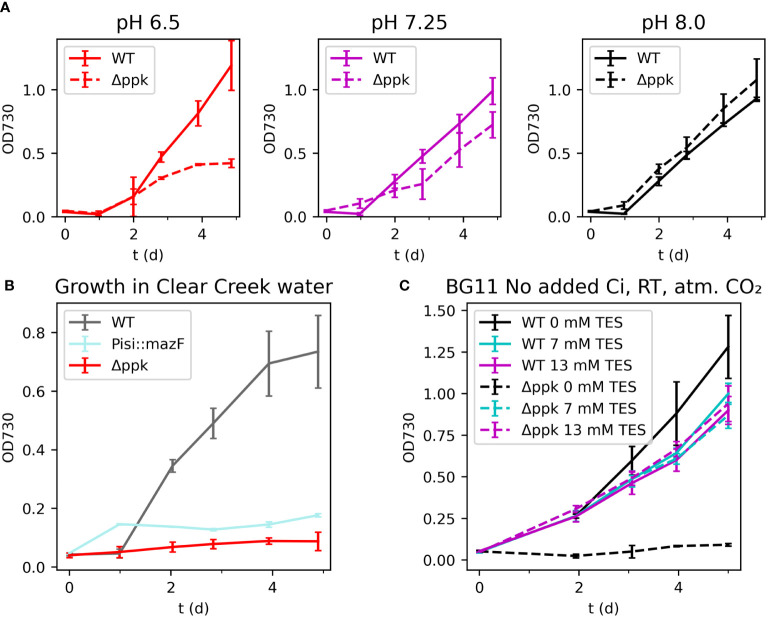
**(A)** Growth in different initial pH media. **(B)** Growth in natural river water. **(C)** Growth in BG11 without added inorganic carbon with different buffer concentrations. The Δ*ppk* mutant is sensitive to a variety of stresses and was uncapable of growth in natural water. **(A)** Growth of Δ*ppk* was reduced in low pH BG11 without supplemented inorganic carbon but similar to WT at higher pH. **(B)** Both *mazF* and Δ*ppk* strains failed to grow in Clear Creek water. **(C)** The Δ*ppk* mutant also failed to grow in BG11 without added inorganic carbon or buffer added. All error bars represent the standard deviation of three biological replicates.

We compared the growth of the WT and Δ*ppk* at different initial pH conditions and simulated spill conditions. The WT and Δ*ppk* mutant were grown with buffer at different pH under ambient room temperature (25°C) and ambient carbon dioxide without added bicarbonate (**Figure 6A**). At a moderately low pH of 6.5, the mutant grew more slowly than WT after two days and ceased growing by day 4 while the WT continued to grow. At pH 7.25, the mutant fell slightly behind the WT after two days but continued growing. At pH 8.0, the mutant and WT grew at similar rates.

As a first step towards a more realistic accidental spill event, we tested the two biocontainment strains in natural water collected from Clear Creek, Colorado, USA in November 2022 (**Figure 6B**). The water was collected, filter-sterilized, and fertilized by addition of K_2_HPO_4_ and sodium nitrate according to the BG11 recipe. In a culture spill scenario, these nutrients would be carried by the spilled medium into the natural water, although likely at lower concentrations. For comparison, a strain containing an active biocontainment module, *mazF*, was generated and tested. This strain utilizes the RNase MazF from *E. coli* driven by the low iron stress-inducible PisiAB promoter and includes the MazE antitoxin expressed from the *E. coli* native *mazE* promotor. In BG11, high iron concentrations repress *mazF*, while in natural water, low iron is expected to induce *mazF* and prevent growth. Cultures were incubated at room temperature (25°C) with ambient carbon dioxide and low light intensity (~30 µmol photons m^-2^ s^-1^). Clear Creek is reported to generally have less than 2 µM iron (and frequently less than 0.5 µM), compared with 22.6 µM in BG11 (Hydros Consulting Inc, 2017). The growth of the *mazF* strain was similar to WT in BG11 (data not shown) but limited in Clear Creek water suggesting that the promoter was activated. Surprisingly, the Δ*ppk* mutant showed no growth in this condition. The reason is unclear, however, additional experiments showed that this strain grew poorly in low inorganic carbon concentrations (**Figure 6C**). The mutant grew similarly to WT in BG11 without added inorganic carbon, pH 8.0, with TES buffer concentrations between 7 and 20 mM. However, the mutant failed to grow when no buffer was present. These observations indicate that a lack of polyphosphate synthesis reduced tolerance of the cyanobacterium to a range of stress conditions.

## 4 Discussion

### 4.1 Polyphosphate is involved in energy management

In this work, we generated a cyanobacterial Δ*ppk* mutant and tested the hypothesis that the mutation could influence energy levels and increase productivity in controlled conditions while also reducing tolerance to stress conditions likely encountered in case of an accidental spill. Our observations agree with an earlier finding in eukaryotic algae, where a mutant of *Chlamydomonas reinhardtii* incapable of polyphosphate synthesis showed higher energy charge in sulfur starvation conditions suggesting that ATP and ADP concentrations could not be adequately controlled in the absence of the polyphosphate synthesis reaction (Sanz-Luque et al., 2020a).

Other research has shown that manipulation of energy management mechanisms can yield faster growth. Synthesis and degradation of molecules such as polyphosphate, glycogen, and sucrose can dissipate energy and therefore represent a maintenance cost that does not contribute to growth. In conditions where specific stresses are not encountered (*i.e.*, in controlled growth settings) the production of these molecules is futile. However, in the natural environment, their continuous synthesis and degradation allows rapid response to fluctuating conditions that cells are likely to encounter. We have recently observed that *Synechocystis* sucrose phosphate synthase knockout and low glycogen mutants also had higher initial energy charges and faster growth compared with WT. Both strains also showed higher maximum photosynthetic capacity in 200 µmol photons m^-2^ s^-1^ light compared with WT (Cantrell et al., 2023). Cano et al. also observed that a glycogen synthesis mutant had a higher initial energy charge than the WT. This mutant also had a reduced lag phase as we observed in the *ppk* mutant (Cano et al., 2018).

Our data indicate that besides the many roles of polyphosphate previously studied such as facilitating phosphate uptake and storage, it also serves energy management. Polyphosphate can be used to either store the chemical energy of ATP to be recovered later by PPK or dissipated via PPX. It is plausible that a futile cycle involving both enzymes operates under a range of light and carbon conditions since both proteins are present in relative abundance (based on proteome data from Jahn et al., 2018). Glycogen and sucrose also have roles in energy management, but there are some differences between polyphosphate, glycogen and sucrose that suggest differences in their functions. Glycogen and sucrose rely on carbon-rich metabolites which may be less available in low carbon conditions. Conversely, polyphosphate may not be a viable option for energy storage when phosphorus is not available. Therefore, the various energy management tools may be utilized based on nutrient availability, to enable energy management under different nutrient conditions.

### 4.2 Higher energy levels trigger faster growth, but are not required to sustain it

The energy charge of the *ppk* mutant was initially higher than that in WT following a shift from our typical low light, autotrophic growth conditions to conditions that produced faster growth in the mutant (**Figures 3**, **4**; glucose and 200 µmol photons m^-2^ s^-1^ light intensity). Later in the log phase of growth, the energy charge was more similar between the strains. In *E. coli*, the energy charge was observed to increase during lag phase. After log phase begins, the energy charge remains constant (Chapman et al., 1971). Similarly, in *Synechocystis*, energy charge increased during lag phase and high energy charge triggered fast growth (Cano et al., 2018). Collectively, the data support the hypothesis that, under these conditions, the lag phase represents a time period for a fresh culture to build up energy charge to reach a threshold that triggers rapid growth. We observed this in both autotrophic and mixotrophic conditions. The Δ*ppk* mutant in this case, as well as the glycogen synthesis mutant in Cano et al. (2018) may reach the threshold more quickly than WT because they lack an ATP sink, thus enabling exponential growth to begin earlier.

### 4.3 Polyphosphate and stress responses

Following P-starvation, we observed a slower return to growth compared with the WT (**Supplementary Figure S5**), similar to what was observed in an exopolyphosphatase (*ppx*) mutant (Hiyoshi et al., 2021). We found that the *Synechocystis* Δ*ppk* deletion mutant could not grow in natural water in a simulated spill test, indicating lower tolerance to stresses. Which specific stress or combination is most responsible for the growth phenotype remains unresolved. One of the stresses is hypo-osmotic stress when sodium concentration in the medium drops from 130 mM to less than 30 mM. Another stress is lower carbon availability (see below). The *ppk* deletion mutant also showed slower growth compared with WT following a shift to a lower pH of 6.5 (**Figure 6**).

Polyphosphate has been implicated in responses to multiple stresses as previously reviewed (Kornberg et al., 1999; Shiba et al., 2000; Achbergerová and Nahálka, 2011; Sanz-Luque et al., 2020b). Resistance to osmotic stress and low pH stress are among the roles found in common across the kingdoms (Xie and Jakob, 2019). Resistance to heat stress, hyperosmotic stress, and oxidative stress has also been associated with polyphosphate in bacteria (Peng et al., 2016; Yoo et al., 2018). Hypo-osmotic stress rapidly induced hydrolysis of polyphosphate in the fungus Neurospora crassa (Yang et al., 1993). In *E. coli*, polyphosphate has been found to influence DNA replication during stress response by inducing DnaA proteolysis (Gross and Konieczny, 2020). In the eukaryotic alga, *Chlamydomonas reinhardtii*, a mutant incapable of polyphosphate accumulation showed slower growth compared to WT when either nitrogen, phosphorus, or sulfur was limiting (Aksoy et al., 2014). Additional study of this mutant showed that polyphosphate synthesis was essential for maintaining ADP when phosphorus was available, but sulfur or nitrogen was unavailable (Sanz-Luque et al., 2020a).

### 4.4 Connections between polyphosphate and low carbon acclimation

Our simulated spill test in natural water involved a sudden shift from 5% CO_2_ atmosphere with supplemented bicarbonate to atmospheric CO_2_ without supplemented inorganic carbon. We observed a growth defect in the Δ*ppk* mutant relative to WT in this test suggesting a connection to carbon uptake for polyphosphate. Further tests showed that growth of the mutant was also inhibited in unbuffered BG11 without added inorganic carbon in atmospheric CO_2_ (**Supplementary Figure S7**).

Gomez-Garcia et al. (2013) previously studied a *ppk* deletion mutant of a thermophilic cyanobacteria *Synechococcus* sp. OS-B’ isolated from a hot spring. Similar to our observations, they found that *ppk* deletion reduces growth in cultures bubbled with air compared to WT and the mutant grew similarly to WT when supplemented with 3% CO_2_. The authors suggested that the mutant lacking polyphosphate is unable to provide energy to the carbon concentrating mechanism. It was also found that transcripts of several protein components of the carboxysome were expressed 50-150 fold higher in the Δ*ppk* mutant compared to WT suggesting that the carboxysome requires polyphosphate for efficient assembly (Gomez-Garcia et al., 2013).

In cyanobacteria, carbon uptake by the bicarbonate transporter is regulated by the ATP : AMP ratio (Selim et al., 2023). Ribosome profiling during acclimation to low inorganic carbon conditions in *Synechocystis* showed increased translation of the *ppk* mRNA and reduced translation of the *ppx* mRNA (Karlsen et al., 2018). Carboxysomes have often been observed to be co-located with polyphosphate granules (Gomez-Garcia et al., 2013). Huokko et al. (2021) found electron-dense granules which were suspected of being polyphosphate adjacent to carboxysomes in *Synechococcus* sp. PCC 7942 (Huokko et al., 2021). Iancu et al. observed close association under electron microscopy between carboxysomes and polyphosphate in three different chemolithoautotrophic bacteria (Iancu et al., 2010). Some images collected showed a regularly repeating lattice at the interfaces (Fraley et al., 2007). Those structures were suggestive of the oligomers in *Pseudomonas aeruginosa* (Ishige et al., 2002) or filaments in *Dictyostelium discoideum* (Gómez-García and Kornberg, 2004) of polyphosphate kinase. A possible reason for colocation was suggested: polyphosphate may be used to maintain pH homeostasis within the carboxysome (Kornberg et al., 1999).

To fully characterize the biocontainment potential of the strain studied here, further work is needed. It is unclear whether the mutation caused cell death or merely prevented growth. Growth tests conducted here may not capture the potential for persistence in the low carbon condition which may eventually allow the strain to re-acquire the *ppk* gene through horizontal gene transfer. This issue may be addressed through further genetic medication to delete the pili genes necessary for DNA uptake (Clark et al., 2018). Future biocontainment assessments should also determine other impacts to natural ecosystems that may arise from a spill of genetically modified microbes.

## 5 Conclusions

Manipulation of energy management mechanisms is a promising strategy to concurrently bolster both biocontainment and productivity in cyanobacteria. This can be achieved by knocking out or knocking down key genes involved in energy dissipating futile cycles such as polyphosphate, glycogen, and sucrose cycles. Such genetic manipulations could lead to higher energy charge, higher growth rate, and higher productivity of target metabolites under lab or industrial conditions while weakening cellular tolerance to variations in natural environment.

## Data availability statement

The datasets presented in this article are not readily available because this work did not generate any large dataset. Requests to access the datasets should be directed to jianping.yu@nrel.gov.

## Author contributions

JS: Data curation, Formal analysis, Investigation, Methodology, Writing – original draft. MC: Methodology, Writing – review & editing. ES: Methodology, Writing – review & editing. HH: Investigation, Methodology, Writing – review & editing. CP: Investigation, Methodology, Writing – review & editing. KC: Writing – review & editing. WX: Writing – review & editing. MG: Funding acquisition, Project administration, Writing – review & editing. JY: Conceptualization, Funding acquisition, Supervision, Writing – original draft, Writing – review & editing.

## Author disclaimer

The views and opinions of the authors expressed herein do not necessarily state or reflect those of the United States Government or any agency thereof. Neither the United States Government nor any agency thereof, nor any of their employees, makes any warranty, expressed or implied, or assumes any legal liability or responsibility for the accuracy, completeness, or usefulness of any information, apparatus, product, or process disclosed, or represents that its use would not infringe privately owned rights.
