# Supplementary material for: Polyphosphate kinase deletion increases laboratory productivity in cyanobacteria
Source: Front Plant Sci. 2024 Feb 7;15:1342496. doi: 10.3389/fpls.2024.1342496 (PMC10879606; doi:10.3389/fpls.2024.1342496)
Supplement: Supplementary file 1 [file DataSheet_1.docx]

Supplementary Material

Polyphosphate kinase deletion increases laboratory productivity and confers effective biocontainment in cyanobacteria

Jacob Sebesta, Michael Cantrell, Eric Schaedig, Harvey J.M. Hou, Colleen Pastore, Katherine J. Chou, Wei Xiong, Michael T. Guarnieri, Jianping Yu*

*** Correspondence:** Jianping Yu: Jianping.yu@nrel.gov

# Supplementary Figures and Tables


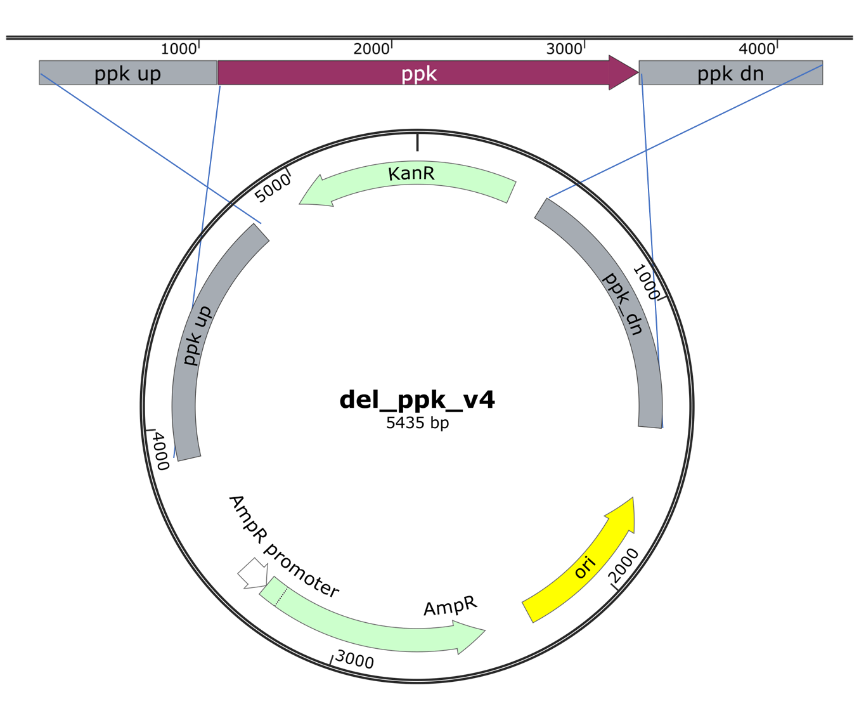


**Supplementary Figure 1.** Plasmid used for generation of *ppk* knockout mutant


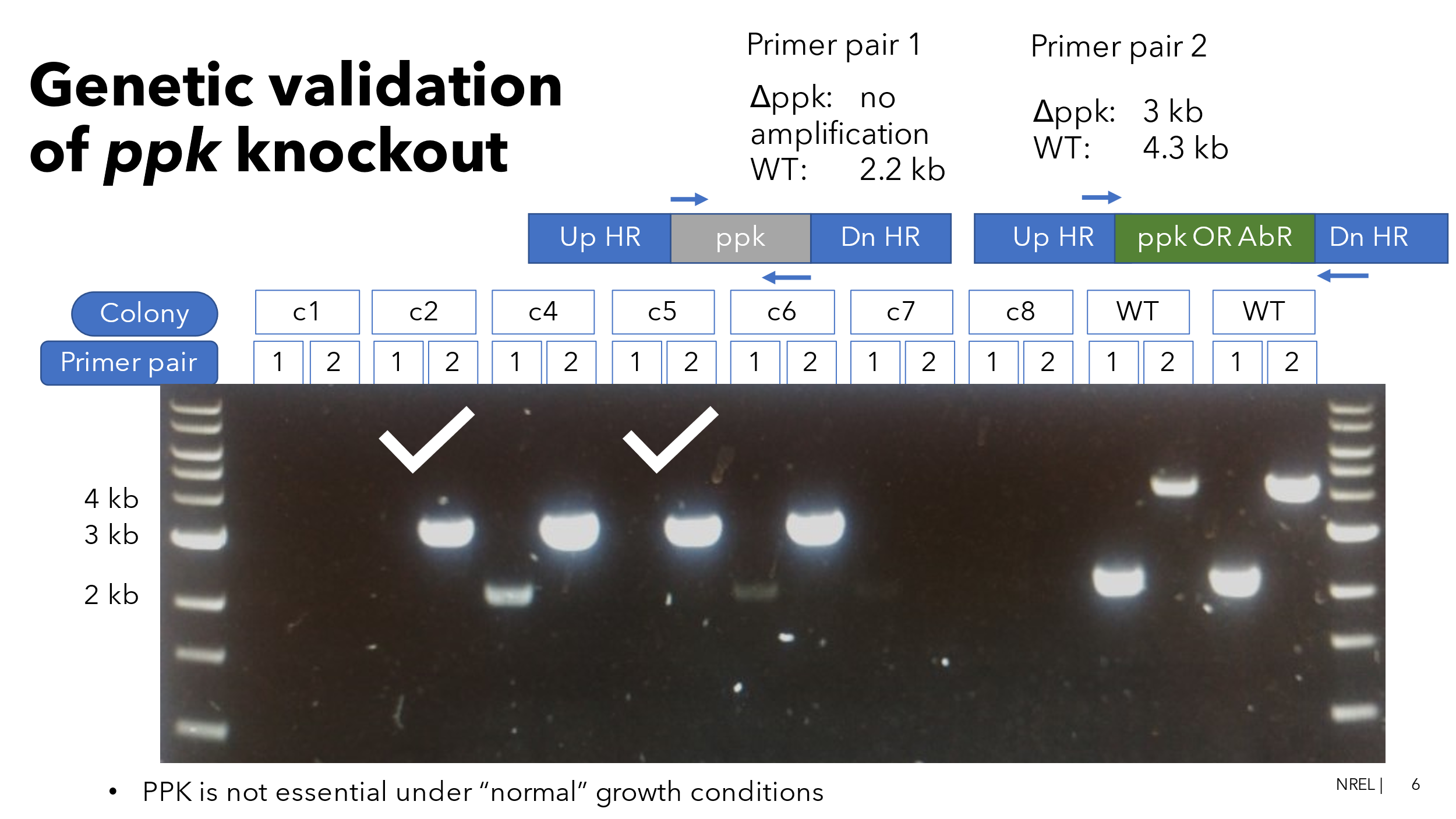


**Supplementary Figure 2.** Genetic validation of *ppk* deletion by colony PCR.

**Supplementary Figure 3.** Phosphate remaining in medium during growth of WT and Δ*ppk.* Error bars represent the standard deviation of three biological replicates

**Supplementary Figure 4.** Growth of WT and Δ*ppk* in BG11 (30 °C, ~40 µmol photons m^-2^ s^-1^ light, 5% CO_2_ atmosphere). All error bars represent the standard deviation of three biological replicates.

**Supplementary Figure 5.** Δ*ppk* is slower to resume growth after 20 µM phosphate supplied to P-starved cultures. All error bars represent the standard deviation of three biological replicates.


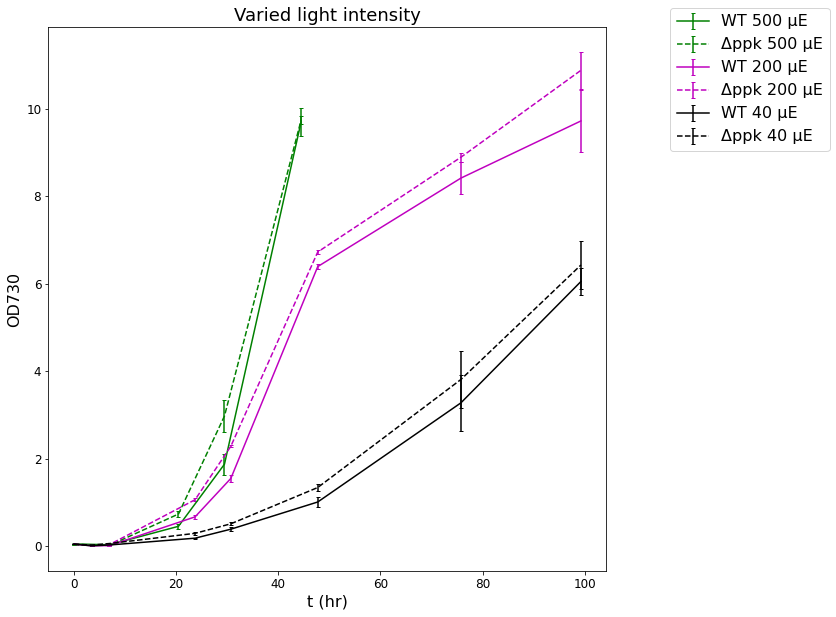


**Supplementary Figure 6.** Comparison of WT and Δ*ppk* growth at different light intensities (30 °C, 5% CO_2_). All error bars represent the standard deviation of three biological replicates.

**Supplementary Figure 7.** Growth of WT and Δ*ppk* at room temperature (25 °C), atmospheric carbon dioxide, in BG11 without buffer or supplemented inorganic carbon. All error bars represent the standard deviation of three biological replicates.


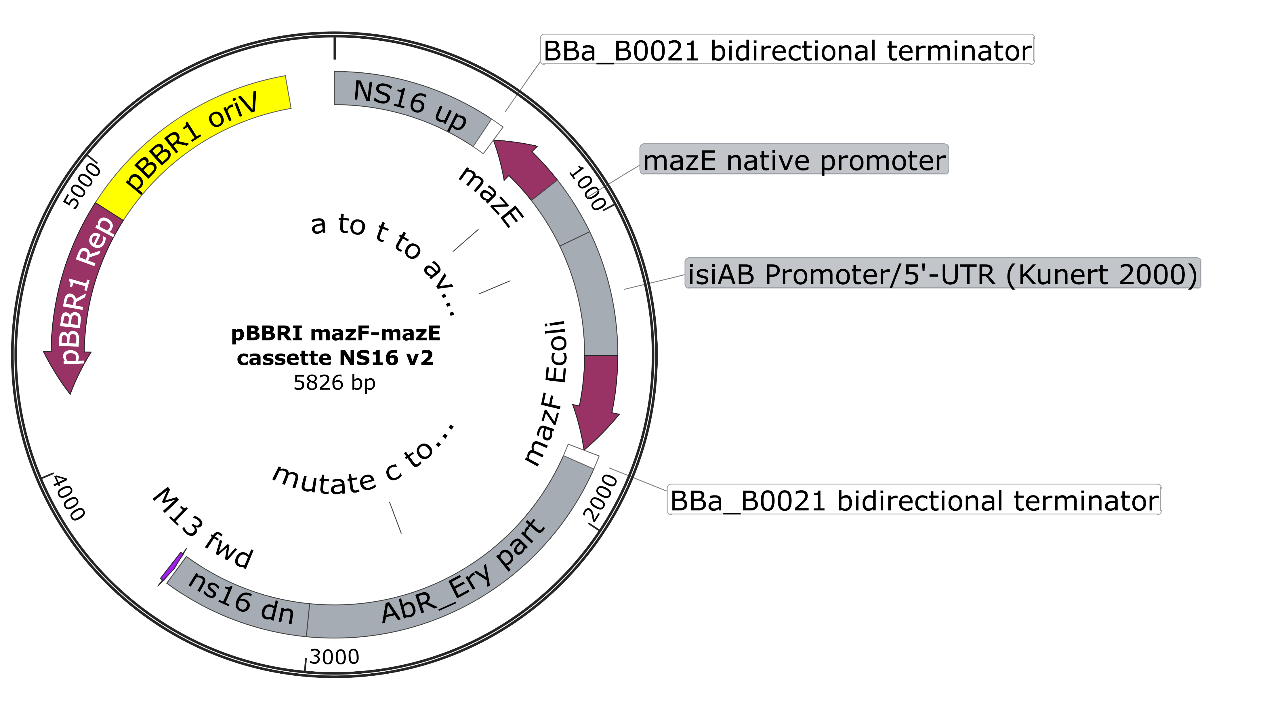


**Supplementary Figure 8.** Plasmid used for *mazF* strain generation


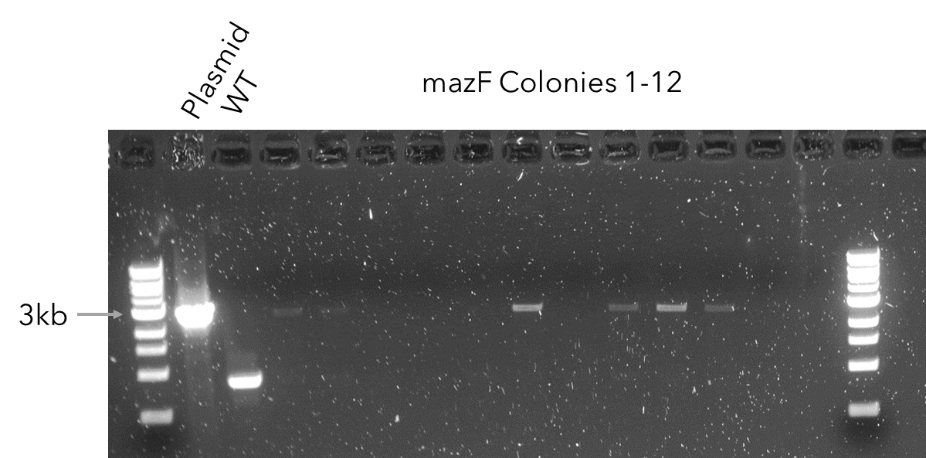


**Supplementary Figure 9.** PCR verification of *mazF* strain. Primers anneal to the Neutral site 16 (NS16) homologous regions (Pinto et al. 2015).

**2 Supplementary Material References**

Pinto, Filipe, Catarina C. Pacheco, Paulo Oliveira, Arnau Montagud, Andrew Landels, Narciso Couto, Phillip C. Wright, Javier F. Urchueguía, and Paula Tamagnini. 2015. “Improving a *Synechocystis* -Based Photoautotrophic Chassis through Systematic Genome Mapping and Validation of Neutral Sites.” *DNA Research* 22 (6): 425–37. https://doi.org/10.1093/dnares/dsv024.
